# Rehabilitation Treatment of a Patient With Total Humeral Endoprosthetic Replacement

**DOI:** 10.7759/cureus.60716

**Published:** 2024-05-20

**Authors:** Naoki Choda, Yoshihiro Kanata, Norihiko Kodama, Saya Iwasa, Takayuki Kawaguchi, Yuki Uchiyama, Hiroyuki Futani, Kazuhisa Domen

**Affiliations:** 1 Rehabilitation Center, Hyogo Medical University Sasayama Medical Center, Tambasasayama, JPN; 2 Department of General Medicine and Community Health Science, Hyogo Medical University Sasayama Medical Center, Tambasasayama, JPN; 3 Department of Physical Therapy, School of Rehabilitation, Hyogo Medical University, Nishinomiya, JPN; 4 Department of Orthopaedic Surgery, Hyogo Medical University, Nishinomiya, JPN; 5 Department of Rehabilitation Medicine, School of Medicine, Hyogo Medical University, Nishinomiya, JPN

**Keywords:** articular range of motion, return to work, chondrosarcoma, rehabilitation treatment, abduction brace, total humeral endoprosthetic replacement

## Abstract

Total humeral endoprosthetic replacement (THR) is a rare surgery for malignant humeral bone tumors. Studies focusing on its surgical methods and functional status are limited. Furthermore, rehabilitation treatment after THR has not been reported. Therefore, this case report aimed to investigate its postoperative rehabilitation treatment and reinstatement. A 69-year-old woman was diagnosed with chondrosarcoma of her left humerus. THR was performed the day following patient admission. The wide resection caused the loss of her left shoulder motor function. She had a left ulnar nerve disorder and carpal tunnel syndrome. Rehabilitation treatments such as joint range of motion training were initiated on postoperative day (POD) 1. We designed a shoulder abductor brace to maintain her left shoulder in an abducted and flexed position so she could use her left hand effectively. The manual muscle testing scores for elbow joint movements gradually improved. On POD47, she was transferred to a convalescent rehabilitation hospital to receive training in activities of daily living and barber work. The patient was discharged on POD107. The Disabilities of the Arm, Shoulder, and Hand score improved from 86.2 (POD7) to 17.2 (POD107). She continued outpatient rehabilitation and reinstated work on POD143. The use of a brace and seamless rehabilitation from the acute phase to convalescence and community-based rehabilitation enabled the patient with THR to return to work. This study suggests that precise assessment of the disorders and consecutive rehabilitation treatment with a brace should be considered after THR.

## Introduction

The humerus is commonly affected by malignant bone tumors, and some patients require radical surgical excision [1]. Limb salvage is more cost-effective than ablative surgery and can improve functional outcomes without worsening life prognosis [2-4]. Options for limb salvage include autografts, alloprosthetic composites, and endoprosthetic replacement [1]. Total humeral endoprosthetic replacement (THR) is the most reasonable reconstruction method for complete humeral excision, allowing relatively fast and good restoration of elbow function. However, it leads to limited active shoulder movements and is a relatively rare surgical operation [5,6].

Several reports have described the surgical method, prognosis, and functional status of patients who underwent THR [1,5-11]. However, the impact of THR on patients’ rehabilitation and lives (e.g., the ability to work) remains unclear. This case report aimed to report the effects of THR on the rehabilitation and daily life of a 69-year-old woman with chondrosarcoma of the left humerus. The findings could help develop approaches for improving the rehabilitation and quality of life of patients with THR.

## Case presentation

History of present illness, past medical history, and social status

A 69-year-old woman was admitted to the orthopedic department of our hospital with a diagnosis of chondrosarcoma of the left humerus. Five months before admission, she experienced left upper arm pain after a bicycle accident. No obvious humeral fractures were observed on radiographs. The pain did not reduce, so she visited the hospital again. Two months before admission, she was diagnosed with chondrosarcoma based on magnetic resonance imaging (MRI) and humerus biopsy.

Comorbidities included hyperlipidemia and left carpal tunnel syndrome. The patient had a history of cholecystectomy and transient ischemic attack. She was taking antiplatelet and antihyperlipidemic drugs with vitamin B12 tablets. The patient’s family history was unremarkable. She was a barber living with her husband and eldest son. Although her husband partially helped with bathing and dressing, she performed other activities of daily living (ADLs), including housework such as cooking and washing, with her left hand as an assistant before admission.

Inspection and staging of the cancer

**Figure 1:**
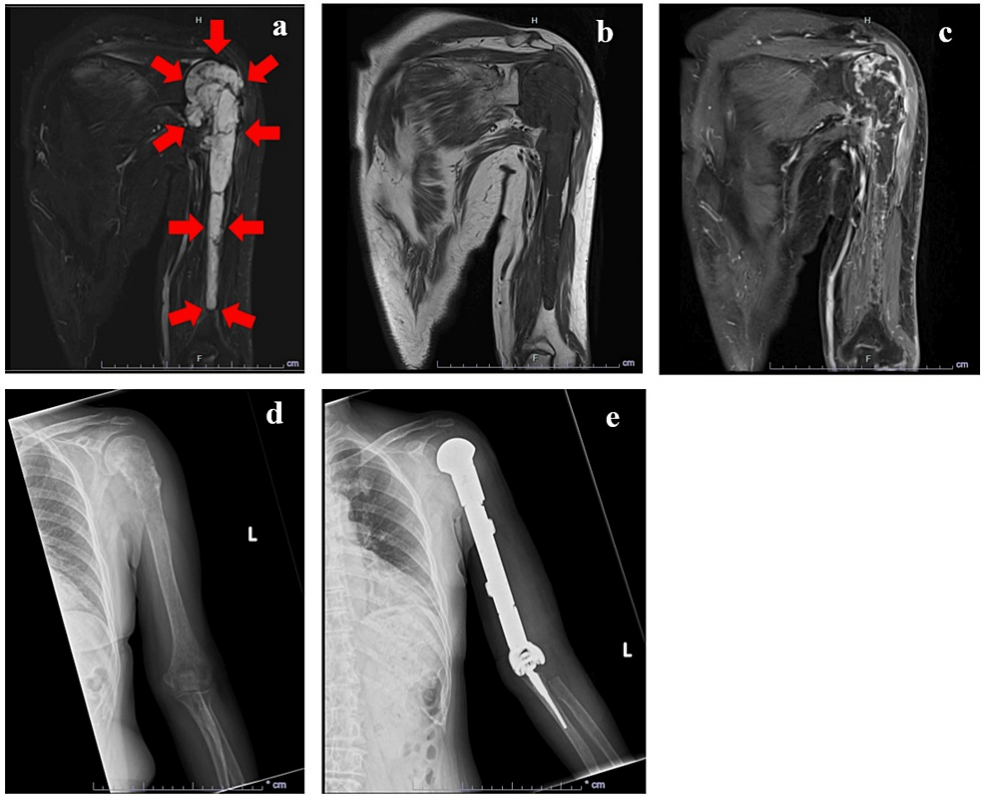
MRI and X-ray images of the left humerus STIR (a), T1-weighted (b), and T1-weighted contrast-enhanced (c) images before surgery and X-ray images before (d) and after surgery (e) are shown. MRI, magnetic resonance imaging; STIR, short τ inversion recovery Arrows indicate the tumor observed from the head of the humerus to the distal four-fifths of the diaphysis.

On MRI, the tumor was observed from the head of the humerus to the distal four-fifths of the diaphysis. It showed high signal intensity on short τ inversion recovery (STIR) images (Figure 1a) and low signal intensity on T1-weighted (Figure 1b) images. T1-weighted contrast-enhanced images revealed heterogeneous enhancement with a surface predominance (Figure 1c). Whole-body positron emission tomography/computed tomography revealed no obvious metastases. Based on the biopsy results, the chondrosarcoma grade was 1 or 2. According to the American Joint Committee on Cancer (AJCC) [12], sarcomas are classified as T2 N0M0G1 (cStage IB) or T2 N0M0G2 (cStage III). X-ray images before and after the surgery are shown in Figures 1d, 1e.

Surgery

The patient provided written informed consent for the surgery and postoperative rehabilitation. She was also provided informed consent for the publication of this report. THR of the left arm was performed on the day after admission (Figure 1e). The Nexel system (Zimmer Biomet, Warsaw IN, USA) was used. The humerus was completely replaced with an implant. The proximal ulna was amputated, and an ulnar graft was inserted. The proximal radius was partially resected. The scapula was intact, and no components were inserted. As the deltoid and most of the rotator cuff were eliminated, shoulder motor function was expected to be lost. Insertions of other shoulder-surrounding muscles, such as the pectoralis major and latissimus dorsi, were cut and not sutured. The brachialis and triceps brachii were ablated from the humerus and sutured to an artificial humeral component. The origin and insertion of the biceps brachii and forearm muscles were considered to be at least partially affected by the surgery. The main trunks of the median, ulnar, and radial nerves were preserved. The chondrosarcoma grade was determined as 1 or 2 based on histopathological examination of the removed humeral component.

Postoperative medical examination and evaluation

Edema occurred in the entire left upper extremity on postoperative day (POD)1. In addition to worsening hypoesthesia in the median nerve region of the left palm, numbness and sensory disturbances were noted on the ulnar side of the left forearm. Manual muscle test (MMT) scores for the left arm were as follows: elbow flexion and extension, 2; supination and pronation, 2; wrist flexion and extension, 3; finger flexion, 3; pollicis abduction, 2; little finger abduction, 0. The functional independence measure (FIM) score was 79 points (motor, 44 points; cognitive, 35 points).

We performed a nerve conduction study and needle electromyography on POD40 (Tables 1, 2). Distal latency in the motor nerve conduction test with median nerve stimulation was delayed on both sides. The motor conduction velocities (MCVs) of the left median and ulnar nerves decreased. We evaluated the MCV of the ulnar nerve between the wrist and below the cubital tunnel because the potential was not observed above the cubital tunnel. Denervation and reduction in the interference pattern were observed at the first dorsal interosseous and flexor carpi ulnaris muscles, although the findings in the C8-paraspinal muscle were almost normal. She was electrophysiologically diagnosed with bilateral carpal tunnel syndrome and a left ulnar nerve disorder between the cubital tunnel and brachial plexus. Furthermore, a conduction block around the cubital tunnel may have occurred. According to the ultrasound scan, no obvious hematoma or artifact compressing the nerves was observed.

**Table 1:** Nerve conduction study on POD40 The motor conduction velocity of the ulnar nerve was assessed between the wrist and cubital tunnel. POD40, postoperative day 40.

	Distal latency (msec)	Amplitude (msec)	Motor conduction velocity (m/s)
Right median nerve	11.2	2.5	61.5
Right ulnar nerve	3.0	10.9	63.9
Left median nerve	9.6	0.31	32.9
Left ulnar nerve	2.9	0.33	33.2

**Table 2:** Needle electromyography study of the left upper extremity on POD40 APB, abductor pollicis brevis; FDI, first dorsal interosseous; FCU, flexor carpi ulnaris; BC: biceps brachii, TC: triceps brachii; C8-paraspinal: C8-Paraspinal erector, IA, insertional activity; Fibs, fibrillation; Psw, positive sharp wave; Fasc: fasciculation, Amp, amplitude; Dur, duration; Poly, polyphase; Int Patt.: interference pattern; MUP, motor unit potentials; POD40, postoperative day 40. Assessment of Poly: 4+ indicated that all MUPs were in polyphase; 0 means the absence of MUPs; 1+, 2+, and 3+ were ranked semiquantitatively between 0 and 4+. Assessment of interference patterns: Normal, many MUPs existed, and the baseline was invisible; 1+, one or two MUPs existed. The 2+ and 3+ groups were ranked semiquantitatively between the 1+ and control groups.

	IA	Fib	Psw	Fasc	Amp (mv)	Dur (ms)	Poly	Int Patt
APB	-	-	-	-	0.3-0.5	5-9	4+	1+
FDI	-	-	+	-	0.3-0.6	3-6	3+	1+
FCU	-	+	-	-	0.3-0.4	5-10	3+	1+
BC	-	-	-	-	0.2	4-6	3+	1+
TC	-	-	-	-	0.4-0.7	4-6	1+	2+
C8-paraspinal	-	-	-	-	0.3-0.5	5-9	0	3+

Based on the International Classification of Functioning, Disability, and Health (ICF), limitations in body functions and structures included shoulder function loss, muscle weakness, and sensory disorders in the left limb, which resulted from THR, left carpal tunnel syndrome, and/or ulnar nerve disorder. However, the patient eagerly wished to return to work as a barber, although her family and friends recommended that she quit the job. The short-term goal was to alleviate the edema and improve the left upper limb function. The midterm goal was independence, and the final goal was to improve housework and work abilities using a brace.

Treatment and outcome

**Figure 2:**
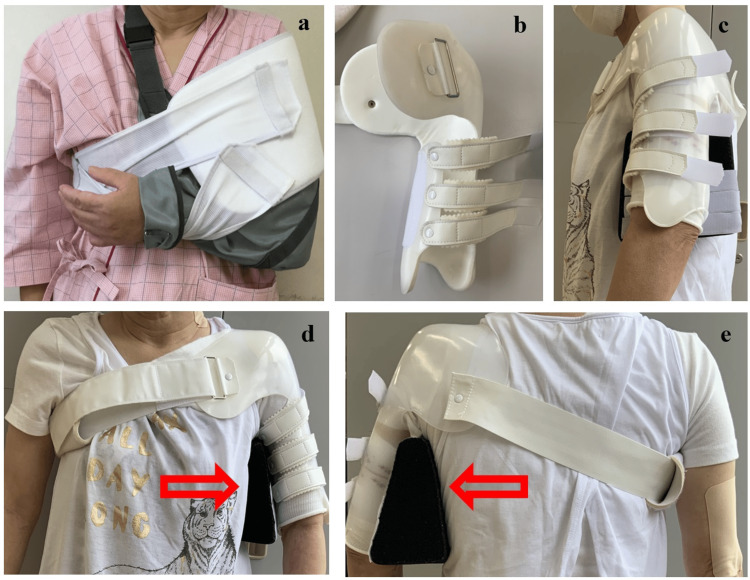
Fixing method for the left shoulder a: Chest band and arm sling. b: Shoulder brace without abduction pillow. c: Side view of the shoulder abduction brace. d: Front view. e: Back view. Arrows indicate abduction pillow.

Acute phase rehabilitation was started on POD1. Physical therapy (PT) was administered to prevent disuse. She performed exercises such as using an ergometer and climbing stairs. To improve elbow, wrist, and finger functions, occupational therapy (OT) was conducted with low-impact joint range of motion (ROM) training and muscle strengthening exercise (MSE) with active assistive movement. An elastic bandage was also applied to reduce edema. In addition, she was instructed to self-train the fingers and wrist in her free time. She was instructed to place the left forearm above her heart when sleeping. Initially, she was instructed to rest the shoulder joint for 24 h and immobilize it with a chest band and arm sling. She could not use her left arm, including her fingers, as an auxiliary hand in daily life (Figure 2a). The orthosis was modified at POD65 after adjusting the size (Figures 2b-2e).

We suggested that the surgeon create a brace allowing the patient to use her left upper limb as much as possible daily. Accordingly, a shoulder abduction brace that fixed the shoulder joint and allowed free movement of the elbow, wrist, and fingers was developed. The brace was designed to set her shoulder at approximately 15° of flexion and 30° of abduction with an abduction pillow attached by velcro. On POD46, the MMT score for elbow joint movement improved to 3, and the FIM score improved to 101 points. The circumference around the metacarpophalangeal joint was 19.5 cm on POD1 and gradually shortened after POD21, measuring 17.5 cm on POD46.

On POD47, the patient was transferred to the recovery hospital, and the edema was unremarkable. She continued ROM training and MSE for the left wrist and elbow joints in addition to ADLs. She was almost independent in ADLs one month after the hospital transfer. At this time, she sometimes needed minimal assistance to shower, dress her upper body, and wear her brace; she found it difficult to pass through the right side of her sleeve and wash the right upper part of her body by herself. By using her left hand as an assistant, her FIM scores were modified and indicated complete independence in eating, grooming, dressing of the lower body, toileting, transfer, and walking. She could wear clothes and shower alone for another 2-3 weeks. To take a shower, she washed her right hand and back using a back brush attached to the wall of the bathroom. She dried her body using a towel placed on a chair.

**Figure 3:**
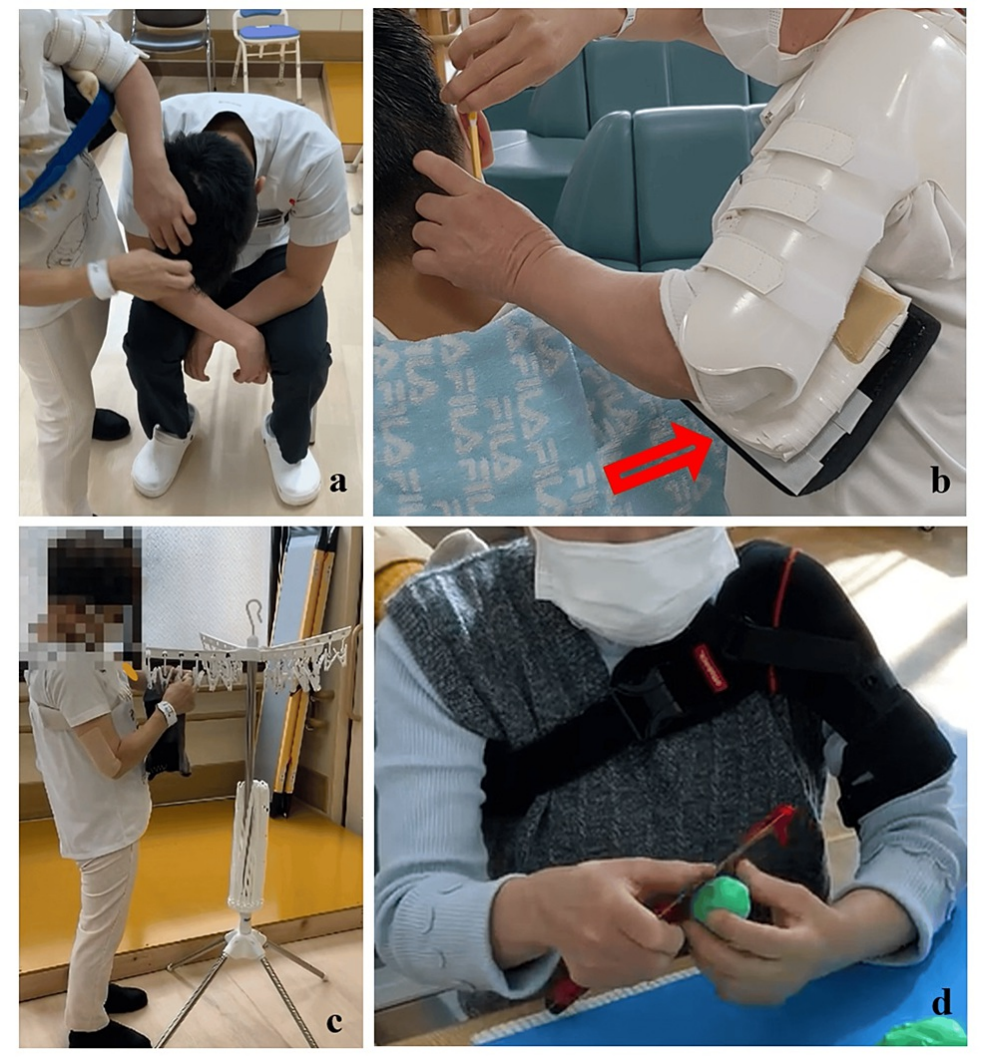
Rehabilitation treatments by OT for returning to barber work and housework a: Washing hair. b: Shaving. c: Hanging laundry. d: Knife handling. OT, occupational therapy Arrow: Additional white cushion to increase the shoulder abduction angle from 30° to 40°.

The orthosis (Kinki-Kishi, Japan) was modified at POD65 after adjusting the size (Figures 2b-2e). Subsequently, she mainly received training for barber work, such as shaving and washing hair, and housework while wearing a brace (Figure 3). She complained of upper limb fatigue at an abduction angle of 30° during shaving, and it was increased to 40° using a cushion (Figure 3b). The flexion angle was optimally set at 15°-30°. The brace did not firmly restrict internal rotation, and the angle was approximately 30°-40°. However, if it was difficult for her to perform tasks at these angles, she was advised to adjust the height and horizontal distance. The main body of the orthosis consisted of polypropylene, which firmly supported and protected the shoulders. However, its stiffness made it difficult for the patient to wear the brace all day. Her return-to-work training was therefore conducted with the brace for 3 h per day. During other times of the day, she wore her Omo Neurexa (Ottobock, Germany), which was made of soft material and can support the arm in a functional position. However, it cannot be attached to an abduction pillow. We advised the patient to place her forearm on the table to keep her shoulders abducted at approximately 15° of flexion and 30° abduction during cooking (Figure 3d). After installing a handrail in the corridor and a bath chair in the bathroom, the patient was discharged home on POD107. Her FIM score was improved to 121 points.

**Figure 4:**
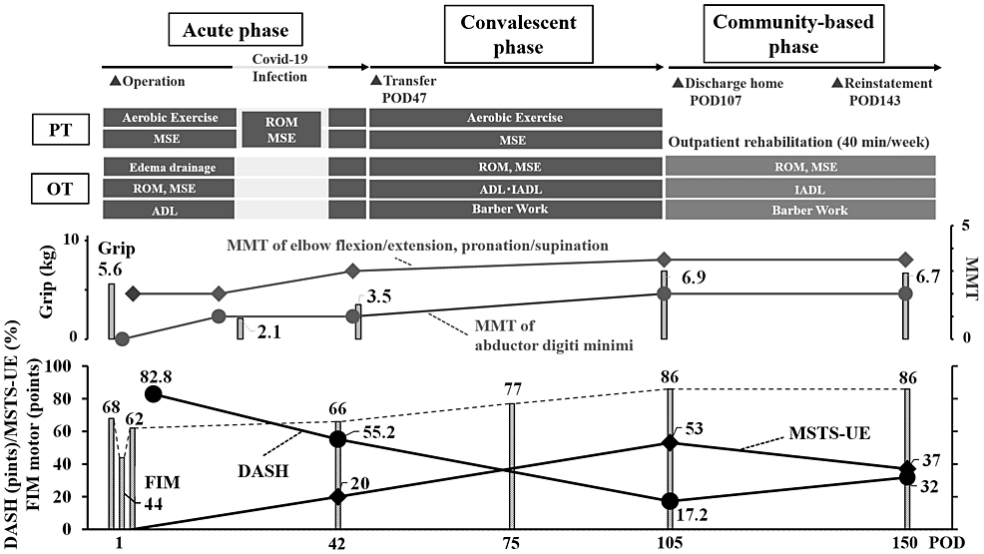
The course of rehabilitation treatment The rehabilitation programs are described in the upper section. Time courses of the left grip (bar) and the MMT of the elbow and forearm (lines) are shown in the middle. FIM (bar), DASH (line with circles), and MSTS-UE (line with diamonds) are shown in the lower part. PT, physical therapy; OT, occupational therapy; MMT, manual muscle testing; FIM, functional independence measure; MSTS-UE, Musculoskeletal Tumor Society scoring system for the affected upper extremities; DASH, disabilities of the arm, shoulder, and hand; ROM, range of motion exercise; MSE, muscle strengthening exercise

Passive elbow flexion was 85° with a 40° flexion contracture on POD3. The ROM improved to 140° of flexion and 5° of flexion contracture on POD105. Supination improved from 80° to 90°, and pronation improved from 25° to 90°. The left grip strength and MMT score gradually improved (Figure 4). We evaluated the FIM score and the Musculoskeletal Tumor Society (MSTS) score for the affected upper extremities (MSTS-UE), as well as the Disabilities of the Arm, Shoulder, and Hand (DASH) score, as shown in the lower part of Figure 4. FIM, DASH, and MSTS-UE scores gradually improved until discharge.

The patient continued outpatient rehabilitation therapy with OT once a week. In addition to barber work and housework training, manual therapy and heat treatment were administered to relieve her stiff shoulders. She put on either an abduction brace or Omo Neurexa in daily life. We confirmed that the braces were used properly. We also confirmed that the patient was voluntarily performing left arm ROM training and MSE, which were taught to her before discharge. She experienced numbness in the median nerve region of her right and left hand. We instructed her to self-train her wrists to relieve the symptoms of carpal tunnel syndrome. She performed housework with the aid of her husband. The owner was contacted directly to check the work environment and the content. After the interview and checking the barber shop owner’s practical skills, she reinstated her work on POD143. However, the DASH and MSTS-UE scores worsened after returning to work. We recommended that she should not work more than twice a week to prevent edema. She was advised to use steps to adjust the height. As she could perform stable voluntary training, the training frequency was lowered from once a week to twice a month after returning to work. Outpatient training was continued until POD230. However, multiple pulmonary metastases were found on computed tomography images, and the patient quit her job on POD270 because of exertional dyspnea. She hoped to spend more time with her family.

Initially, the patient was disappointed with shoulder function loss and nerve disorders. She was also at a loss due to the lack of information on rehabilitation treatment for THR. Unfortunately, she eventually had to quit the job as she expressed great satisfaction after returning to work.

## Discussion

Summary of the case

This case report is about a female barber who underwent THR and successfully returned to work after rehabilitation using a shoulder abductor brace. THR is a rare surgery. To our knowledge, this is the first study to describe a series of rehabilitation treatments after THR. The characteristics of the case are as follows. First, the rehabilitation program was designed with the precise assessment of her disorders and appropriate goal setting. Second, consecutive rehabilitation treatments, including return to work, were performed from the acute phase to the convalescent and community-based stages. Finally, a brace that could keep the shoulder abducted greatly enhanced upper extremity function and activity.

Overall survival, mechanical survival, and complications of THR

Based on a systematic literature review, the chondrosarcoma prognosis depends on many factors, of which tumor grade is one of the most important [13]. The reported five-year survival rate for grade 1 and 2 lesions ranges from 82% to 99% and 63% to 92%, respectively. Our patient had grade 1 or 2 chondrosarcoma, with a five-year survival rate of >63% at the time of surgery.

Several studies have reported the long-term survival and outcomes of THR [1,5,7-11]. The mean follow-up period ranged from 41 to 148 months, and the five-year survival rate was 60%-100%. The 10-year implant survival rate ranged from 65% to 100% [1,7,8]. Symptomatic shoulder instability is a common reason for revision. Commonly reported complications of THR include shoulder instability, loosening of the ulnar component, radial nerve palsy, and infection [7]. The complications in our case were edema, ulnar nerve palsy, and worsening of left carpal tunnel syndrome. Peripheral edema is a risk factor for carpal tunnel syndrome [14], and exacerbation may be an indirect effect of surgery. The patient was electrophysiologically diagnosed with a left ulnar nerve disorder between the cubital tunnel and the brachial plexus. Left ulnar nerve disorders may be caused by surgical techniques and/or postoperative edema. Our patient presented with numbness in the right palm and was electrophysiologically diagnosed with right carpal tunnel syndrome. This is probably because the burden on the right hand increased.

Functional prognosis of THR

Attempts have been made to establish a stable glenohumeral joint by reattaching the rotator cuff, deltoid, and short head of the biceps tendons. Kotwal et al. reported that the mean active shoulder abduction was 12.5°, and flexion was 15° [1]. MMT of shoulder and elbow movements has not been reported previously. Because of the wide resection, the motor function of the shoulder was lost in our case. Conversely, grip strength and the MMT score for elbow motion were gradually improved up to POD105. The MMT score was not 5, possibly because the origin and insertion of the forearm muscles were at least partially affected by the surgery. They also reported that the mean elbow flexion was 103.5°, with a 30.5° flexion contracture [1]. The elbow ROM in our case was almost normal after POD105 and was better than that reported in a previous study. The difference could be attributed to ROM training in the early phase in our case.

Reported MSTS-UE scores range from 72% to 83% [1,5,7-11]. The MSTS-UE score in our case was 37%, lower than those reported in previous studies. One reason for this difference is shoulder function loss. All deltoid and most rotator cuff muscles were eliminated. Other reasons included left ulnar nerve disorder and worsening of carpal tunnel syndrome. Previous reports assessed upper limb function and quality of life using MSTS-UE. Although it is a quick evaluation method, it fails to assess specific daily activities of the upper extremities. Thus, we also evaluated the DASH score, a commonly used measure of symptoms and functional status of the upper extremities [15]. Unlike MSTS-UE, it evaluates both hands. Our patient had bilateral carpal tunnel syndrome, and the DASH scores may more precisely reflect daily living abilities. Both MSTS-UE and DASH scores improved over time and were good at the time of discharge. However, both scores decreased after returning to work, probably because of muscle fatigue and worsening of carpal tunnel syndrome.

Rehabilitation treatment for THR

No previous studies have reported rehabilitation treatment after THR. THR is characterized by total shoulder arthroplasty (TSA) and total elbow arthroplasty (TEA). Regarding TSA, several studies have reported rehabilitation treatments [16-21]. It is important to strengthen the remaining muscles, such as the deltoid, and protect the joint. While 4-6 weeks of immobilization is often recommended postoperatively, recent prospective studies have shown that early motion can improve patient-reported outcome scores with low complication rates [19]. TSA is achieved by implanting components into the humerus and the scapula. However, the components of THR are not usually incorporated into the scapula, making it difficult to achieve stability and smooth shoulder movement. In addition, the muscles surrounding the shoulder are often less preserved in THR than in TSA. In reverse TSA [16], active forward elevation has been reported to be >100°. No studies have reported rehabilitation treatments after TEA.

Edema negatively impacts joint ROM, strength, and hand function [22]. Here, we addressed edema using commonly used treatments such as elevation, exercise, and compression. The condition gradually improved from POD21 and was not notable after POD46.

Nagy et al. determined the optimal position of shoulder arthrodesis for pain and function as follows: 15°-30° of flexion, 35°-45° of abduction, and 30°-40° of internal rotation [23]. Accordingly, our brace was used at approximately 15°-30° of flexion and 30°-40° of abduction.

A limitation of this study is that we encountered only one case of rehabilitation treatment for THR. Nevertheless, we described the case in detail. We believe that a brace, continuous rehabilitation from the acute phase to convalescence, and community-based rehabilitation enabled the patient to return to work despite shoulder motor function loss and nerve disorders.

**Figure 5:**
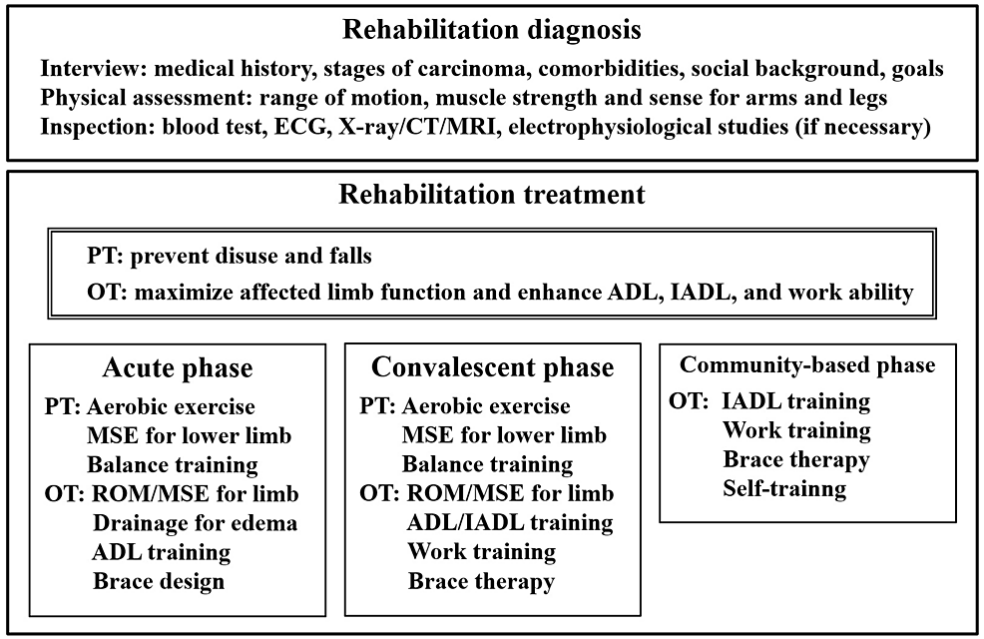
Proposal for effective rehabilitation treatment for THR Representative items of rehabilitation diagnosis and treatment are presented. Convalescent-phase rehabilitation and work training are unnecessary if the patient does not hope to work. Evaluation of the unaffected limb is also crucial because of its increased burden. ROM, range of motion; MSE, muscle strengthening exercise; ECG, electrocardiogram; MRI, magnetic resonance imaging; CT, computed tomography; PT, physical therapy; OT, occupational therapy; ADLs, activities of daily living; IADLs, instrumental activities of daily living

Based on our experience, we propose the following rehabilitation treatments for THR. First, information such as tumor stage, comorbidities, social status, and hopes should be collected. For physical examination, ROM and MMT scores for the affected and unaffected sides should be assessed. After assessment of disorders and goal setting, OT should be prescribed. DASH, MSTS, and FIM scores are appropriate indicators of the treatment response. A shoulder abductor brace can maintain a functional position and enhance upper limb function. Rehabilitation treatment should be initiated in the acute phase and continued through the convalescent and community-based phases, as illustrated in Figure 5.

## Conclusions

We reported a case involving a female barber who underwent THR and successfully returned to work. This report provides insight into the development of rehabilitation strategies for future patients who undergo THR. We believe that a precise assessment of the disorders and consecutive rehabilitation treatment with a brace should be considered after THR.
